# Bilateral Ureteral Insertion Into the Bladder Diverticula Caused by Prostatic Enlargement: A Complex Surgical Scenario

**DOI:** 10.7759/cureus.86895

**Published:** 2025-06-27

**Authors:** Fadhel Yusuf, Amal Hayat, M. Zaid Jarai, Amr Elmekresh, Ayman AlYammahi, Tariq Abdul Hamid, Abdulmunem Alsadi, Elham Mahjoor Azad, Fariborz Bagheri

**Affiliations:** 1 Urology, Dubai Hospital, Dubai, ARE; 2 Urology, Dubai Health, Dubai, ARE; 3 Surgery, Dubai Hospital, Dubai, ARE; 4 Family Medicine, Dubai Health, Dubai, ARE

**Keywords:** bladder diverticula, hematuria, simple prostatectomy, ureteral reimplantation, urinary tract obstruction

## Abstract

Bilateral ureteral insertion into the bladder diverticula is an extremely rare condition, particularly when acquired secondary to longstanding bladder outlet obstruction due to prostatic enlargement.

We present a complex surgical management of a 72-year-old male with a history of diabetes mellitus and hypertension, who presented with severe lower urinary tract symptoms, gross hematuria, and clot retention. Imaging revealed marked prostatic enlargement and two large bladder diverticula arising from the right and left lateral bladder walls. During the planned open simple transvesical prostatectomy and bilateral bladder diverticulectomy, both ureters were unexpectedly found to insert into the diverticula. Consequently, the intraoperative plan was modified to include bilateral ureteral reimplantation.

This case underscores the diagnostic and surgical challenges in managing bladder outlet obstruction complicated by bilateral ureteral insertion into the bladder diverticula. It highlights the importance of individualized surgical planning in managing such unique urological anomalies.

## Introduction

Benign prostatic hyperplasia (BPH) is a common condition characterized by nonmalignant enlargement of the prostate gland, affecting many elderly men [1]. The enlargement causes urethral compression, leading to bladder outlet obstruction and lower urinary tract symptoms (LUTS) [2].

If untreated, BPH can result in complications such as recurrent urinary tract infections, bladder stones, gross hematuria, bladder diverticula, increased bladder wall thickness, and renal impairment [3].

Surgical treatment is recommended for severe symptoms, complications, or failed medical therapy. Transurethral resection of the prostate (TURP) is the standard for prostate volumes between 30 and 80 grams [4], while open simple prostatectomy is preferred for significantly enlarged prostates (>100 g) or when complications are present [5].

Bladder diverticula alone are not always an indication for surgery; however, when associated with recurrent infections or urinary stasis, surgical excision becomes necessary. In this case, the patient presented with significant prostatic enlargement and bilateral bladder diverticula, with both ureters inserting into the diverticula, a rare anatomical variant. This complex scenario necessitated a tailored surgical approach involving open simple prostatectomy, bilateral diverticulectomy, and bilateral ureteral reimplantation. The diverticula likely developed as a result of chronically increased intravesical pressure due to longstanding BPH, contributing to urinary stasis and recurrent infections, and thus required definitive surgical management.

## Case presentation

Patient background

A 72-year-old male with a history of diabetes mellitus and hypertension was referred from another hospital after presenting with gross hematuria, a drop in hemoglobin, and clot retention. At the primary hospital, a contrast-enhanced CT scan was performed; however, only the report was available, as the actual images were not provided. The report described a grossly distended urinary bladder extending up to the level of the umbilicus, with prominent trabeculations and multiple diverticula, the largest measuring 9 × 6 cm. Mild right renal pelvic dilatation was also noted. Incomplete contrast filling of the bladder was observed, likely due to overdistension at the time of imaging. Following the scan, the patient was catheterized with an 18 French silicone Foley catheter and subsequently referred to our tertiary hospital for further evaluation and management.

Initial management

At presentation, the patient was in significant distress, complaining of abdominal pain and dysuria. The existing 18 French silicone catheter had become blocked with clots due to persistent hematuria. Bedside ultrasound showed a grossly distended bladder filled with clots and multiple diverticula. Using a 28 French rectal tube, approximately 500 ml of clots were evacuated. A 24 French three-way Foley catheter was then inserted, and continuous bladder irrigation was started. Mild hematuria resolved within the first hour.

Diagnostic findings and clinical progression

**Figure 1:**
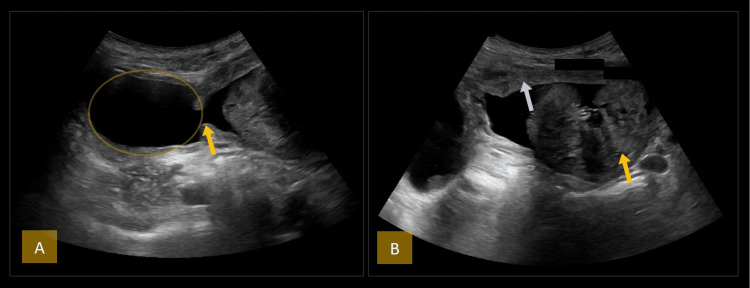
Ultrasonographic evaluation of the bladder and prostate. (A) The yellow arrow indicates the neck of the bladder diverticulum. The yellow circle highlights the right lateral wall bladder diverticulum. (B) The yellow arrow highlights a markedly enlarged median prostatic lobe protruding into the urinary bladder. The white arrow points to diffuse bladder wall thickening consistent with bladder outlet obstruction.

At our facility, departmental ultrasound of the kidneys, ureters, and bladder (KUB) demonstrated multiple bladder diverticula and a markedly enlarged prostate measuring 160 cc, with a prominent median lobe (Figure 1).

**Figure 2:**
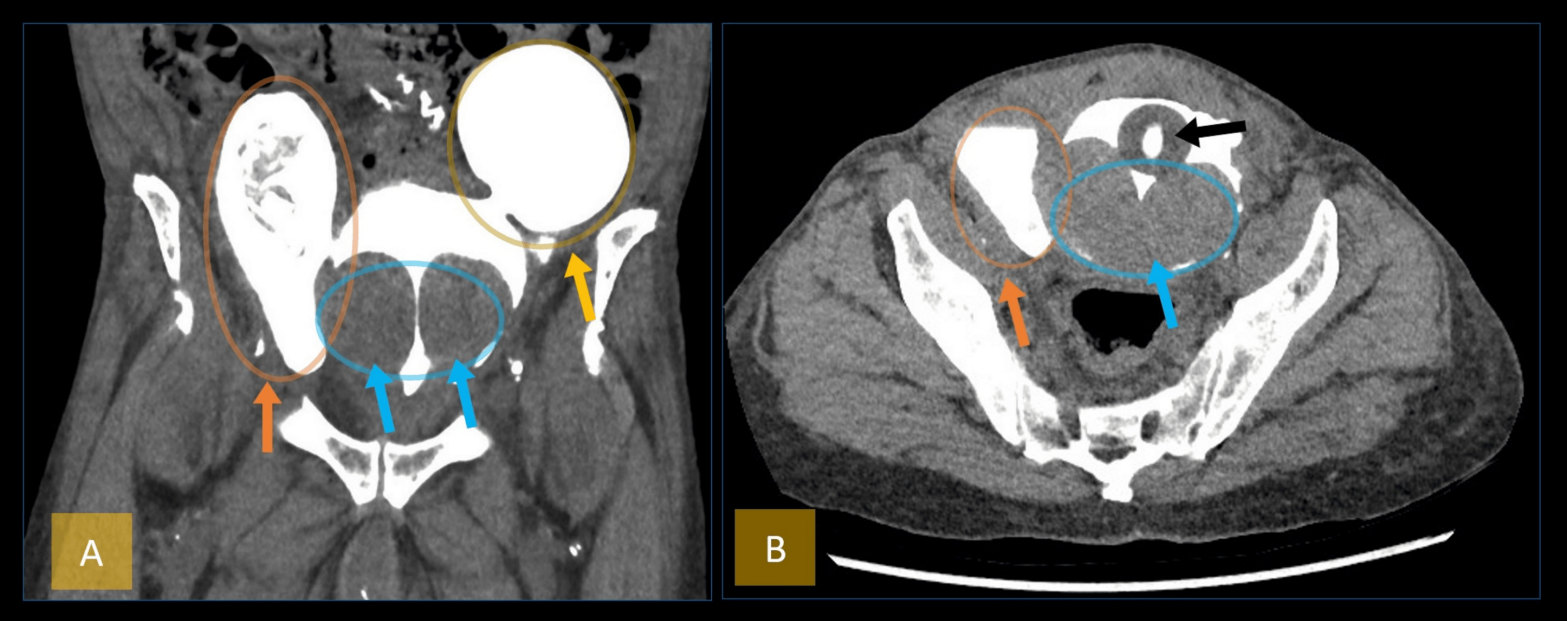
CT of the kidney, ureter, and bladder without contrast with retrograde cystogram demonstrating bilateral bladder diverticula and prostatic enlargement. (A) Coronal view: The orange arrow and circle highlight the right lateral bladder wall diverticulum. The yellow arrow and circle indicate the left lateral bladder wall diverticulum. The blue arrow and circle show the markedly enlarged median prostatic lobe protruding into the bladder. (B) Axial view: The orange arrow and circle mark the right lateral diverticulum. The Foley catheter balloon is identified by the black arrow. The blue arrow and circle point to the markedly enlarged median prostatic lobe protruding into the bladder, consistent with bladder outlet obstruction.

A non-contrast CT of the KUB with retrograde cystogram was done to rule out any bladder injury, which was confirmed to be absent. It confirmed the presence of large bilateral bladder diverticula and a huge median prostatic lobe protruding into the bladder (Figure 2).

Endoscopic evaluation

**Figure 3:**
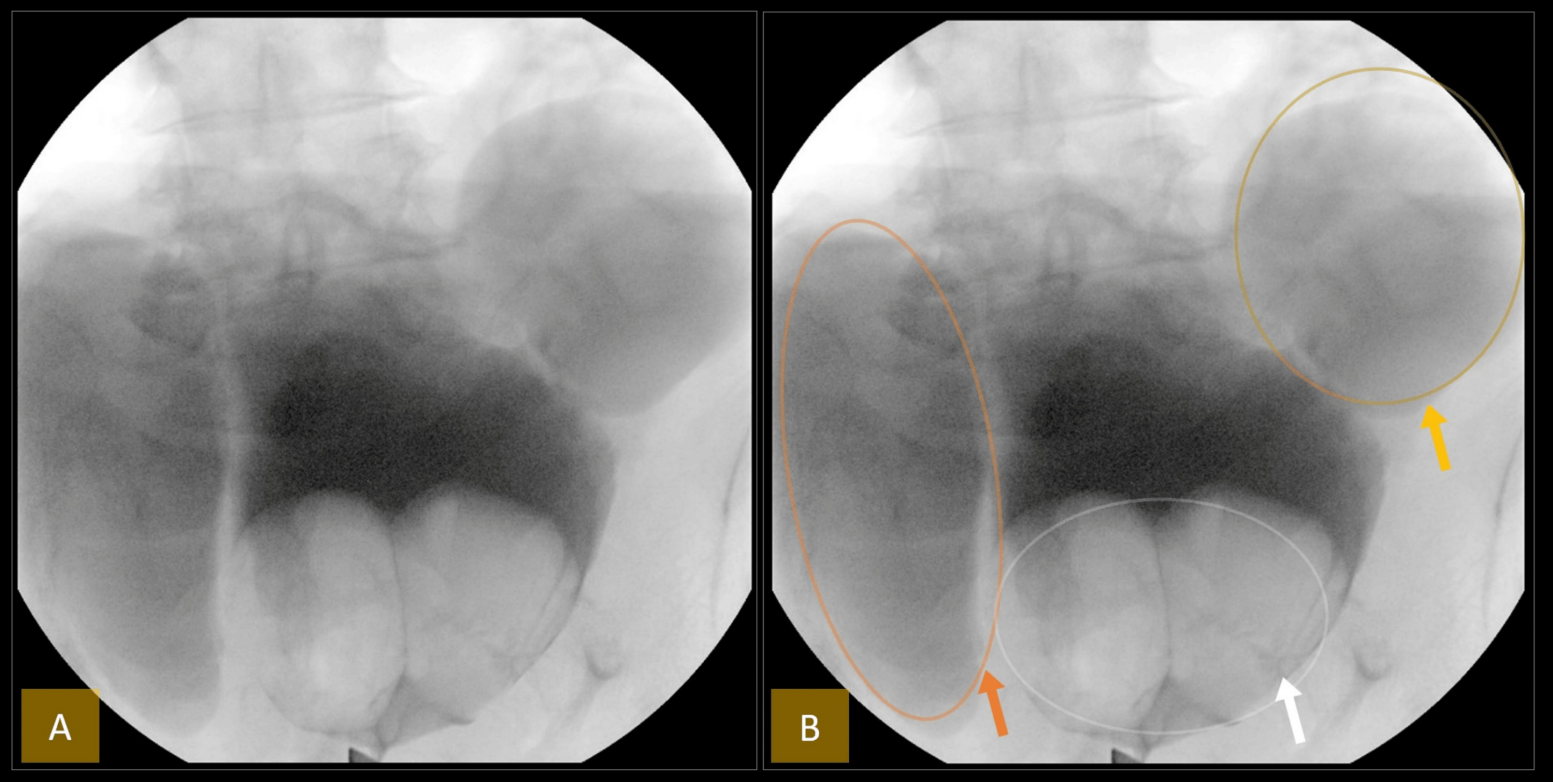
Intraoperative cystogram demonstrating bilateral bladder diverticula and prostatic median lobe enlargement. (A) Intraoperative cystogram showing large bilateral bladder diverticula and an enlarged prostatic median lobe without annotations. (B) Same image with markers: The orange arrow and circle highlight the right lateral wall bladder diverticulum. The yellow arrow and circle indicate the left lateral wall bladder diverticulum. The white arrow and circle highlight the markedly enlarged median prostatic lobe protruding into the urinary bladder.

Due to persistent hematuria, cystoscopy was performed, revealing markedly enlarged prostate lobes protruding into the bladder. The scope could not reach the necks of the diverticula on either side due to the obstructing median lobes. An intraoperative cystogram using fluoroscopy revealed large bilateral bladder diverticula (Figure 3). The bladder mucosa appeared unremarkable, and the ureteral orifices were not visualized, likely obscured by the enlarged prostate lobes.

Surgical intervention

Given the presence of a significantly enlarged prostate (160 cc) and large bilateral bladder diverticula, an open transvesical simple prostatectomy with bilateral bladder diverticulectomy was planned. This approach was favored as it provided optimal exposure for complete excision and allowed flexibility for reconstruction in the event of intraoperative complexities.

Intraoperatively, both ureters were unexpectedly found to insert directly into their respective diverticula. The diverticula were carefully excised while safeguarding the ureters, which were divided and spatulated bilaterally. Meticulous dissection was required to isolate and preserve the ureters during diverticulectomy. A tension-free, watertight ureteral reimplantation was performed to minimize the risk of postoperative complications such as ureteral stricture or reflux nephropathy.

**Figure 4:**
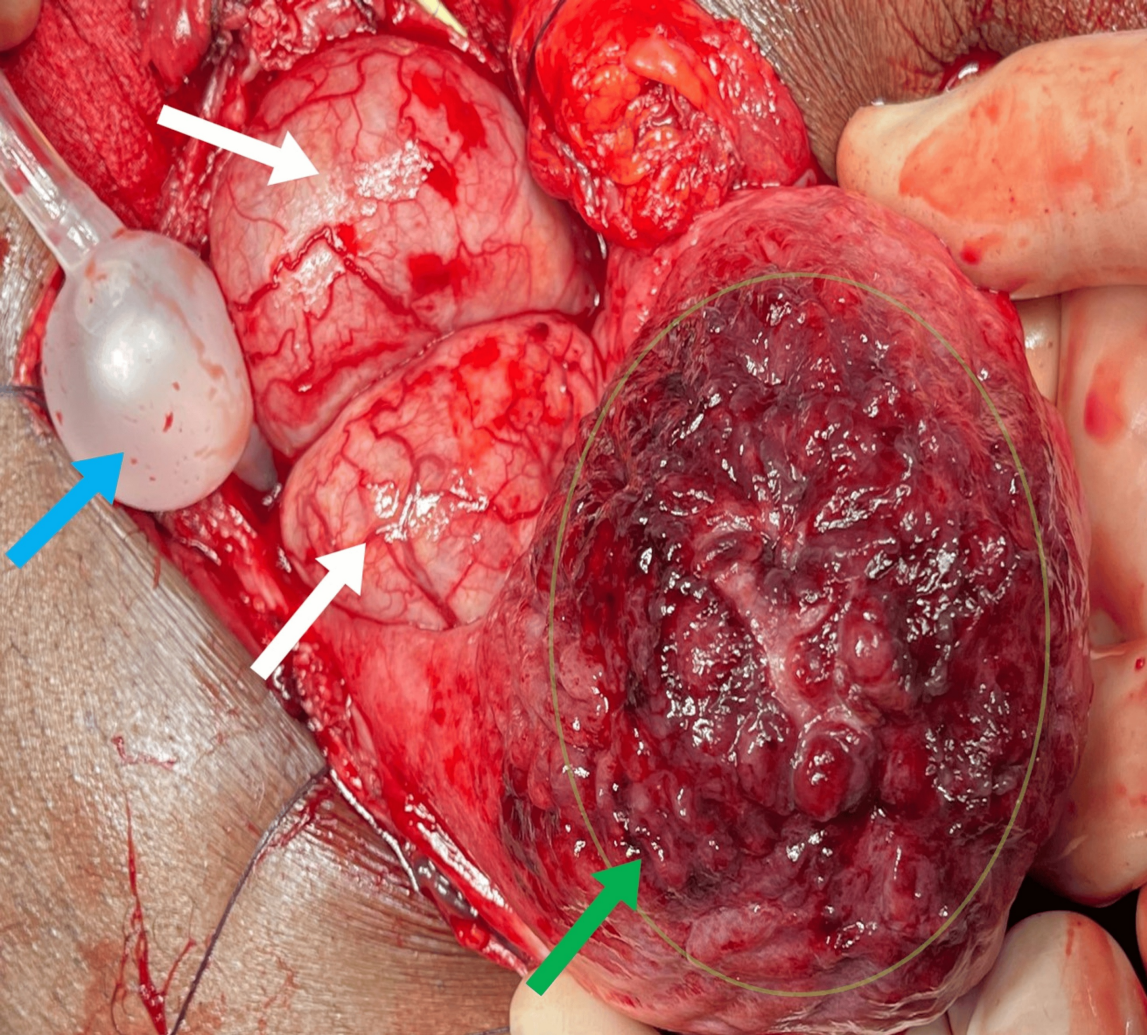
Intraoperative image during transvesical prostatectomy. Intraoperative view of the bladder interior during transvesical prostatectomy. The green arrow and circle highlight the erythematous changes in the bladder dome mucosa, from which a biopsy was taken. White arrows point to the markedly enlarged prostate protruding into the bladder. The blue arrow indicates the Foley catheter balloon.

After the excision, the bladder dome mucosa exhibited erythematous changes but no bleeding. A biopsy sample was taken for histopathology study, which came back negative for malignancy (Figure 4).

**Figure 5:**
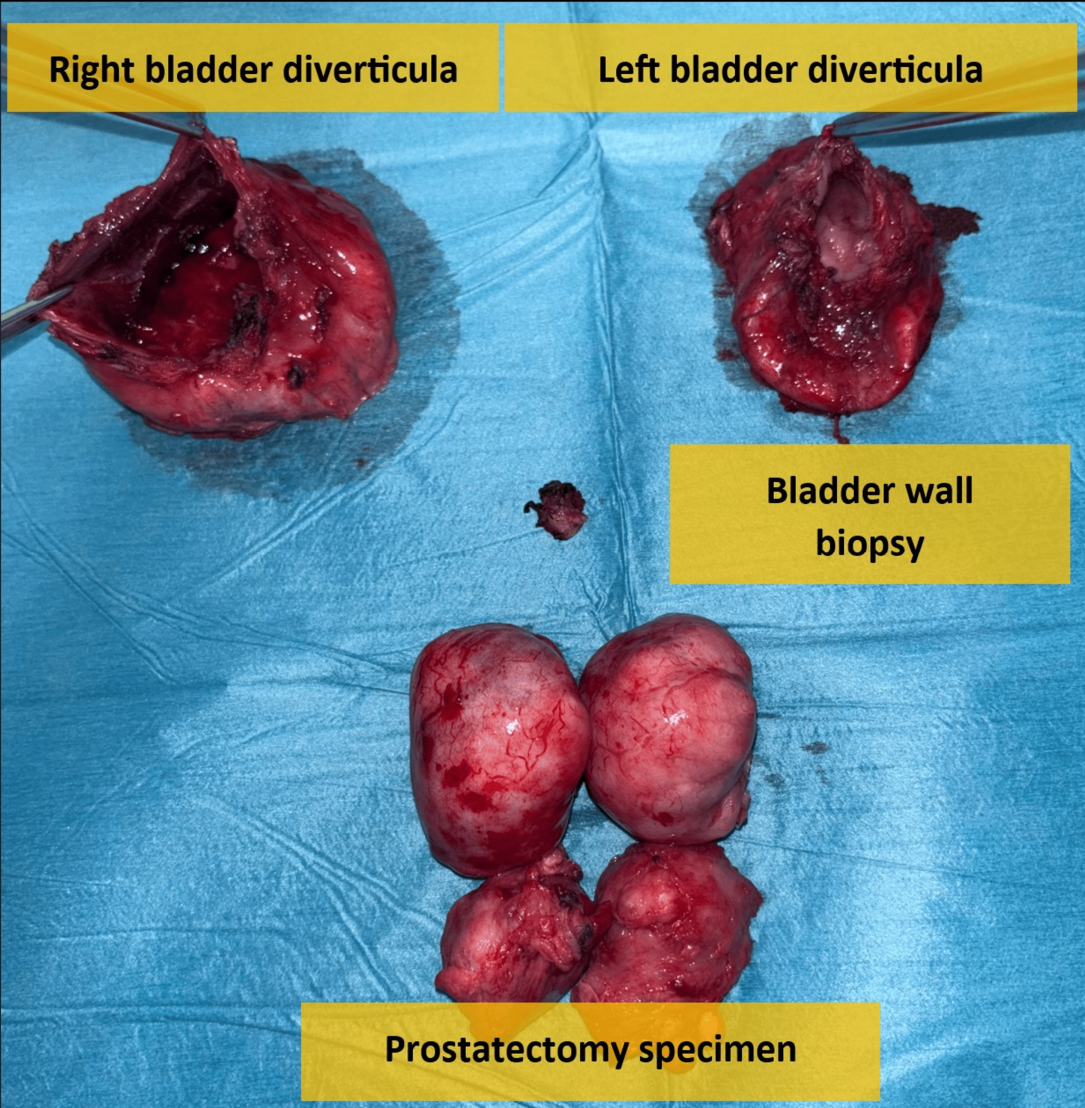
All specimens (both bladder diverticular wall, prostatic lobes, and bladder biopsy).

A tension-free, water-tight ureter reimplantation was performed along with Freyer's prostatectomy. This involved a circumferential incision of the bladder mucosa around the median lobe, the identification of the plane between adenoma and prostate capsule, and the enucleation of the lobes under digital guidance. The bladder was tested for leaks with a 200 ml saline infusion, showing no leaks or bleeding. A perivesical drain was inserted and fixed. All specimens (both bladder diverticular wall, prostatic lobes, and bladder biopsy) were sent to histopathology studies (Figure 5).

Histopathology of both bladder diverticula showed fibrous tissue with congestion and surface erosion, with no evidence of malignancy. The prostate tissue was consistent with benign prostatic hyperplasia and was also negative for malignancy. Similarly, the bladder biopsy revealed no malignant features.

Postoperative course

The patient showed significant postoperative improvement. Clear urine output of 2 liters was observed from the urethral Foley catheter, with minimal drainage from the perivesical drain, which was subsequently removed. The patient was discharged and later underwent successful bilateral ureteral stent removal and bilateral retrograde pyelography, which confirmed the absence of urine extravasation.

## Discussion

Bladder diverticula, frequently associated with BPH, are pouch-like evaginations of the bladder wall that can lead to significant complications [6]. This case illustrates the intricate relationship between bladder diverticula and BPH, highlighting the need for a comprehensive understanding of both conditions for effective management.

Bladder diverticula are classified as either congenital or acquired. Congenital diverticula are typically associated with conditions such as posterior urethral valves or neurogenic bladder, while acquired diverticula most often result from bladder outlet obstruction, commonly due to BPH [6].

The size of bladder diverticula plays a crucial role in the risk of acute urinary retention (AUR) in patients with BPH. Studies suggest that diverticula larger than 5.15 cm significantly increase the risk of AUR, with a sensitivity of 73% and specificity of 72% [7,8]. In this case, the presence of a large diverticulum measuring 9 × 6 cm likely contributed to the patient’s symptoms.

Bladder diverticula may also lead to urinary stasis, stone formation, recurrent urinary tract infections, and, in rare cases, malignancy [6,8]. Additionally, large diverticula can compress adjacent pelvic veins, increasing the risk of deep vein thrombosis and pulmonary embolism [9].

Surgical intervention is often required when bladder diverticula coexist with BPH, especially in the presence of complications or unusual anatomy. In this case, an open prostatectomy and bilateral bladder diverticulectomy were performed, along with bilateral ureteral reimplantation due to the ureters opening directly into the diverticula, an extremely rare anatomical variant.

Although minimally invasive techniques, such as transurethral laser enucleation of the prostate combined with laparoscopic diverticulectomy, are effective in many cases [10], they were deemed unsuitable in this case due to the patient’s complex anatomy and the large size of the diverticula. Open surgery provided better access, precision, and control, which were essential for the successful outcome.

## Conclusions

This case underscores the complex interplay between bladder diverticula and significant prostatic enlargement, illustrating a rare scenario in which both ureters were inserted into the diverticula. Successful management through combined prostatectomy, diverticulectomy, and bilateral ureteral reimplantation highlights the importance of a personalized, multidisciplinary approach. Meticulous intraoperative planning and careful postoperative care were key to achieving a favorable outcome. This report adds valuable insight into the surgical management of rare and anatomically complex urological cases, reinforcing the need for individualized treatment strategies and interdisciplinary collaboration.
